# Supplementary material for: Dual phase high temperature Si3N4/Al(Ti)N films with tunable thermal conductivity
Source: Nat Commun. 2025 Dec 22;16:11555. doi: 10.1038/s41467-025-67582-y (PMC12749134; doi:10.1038/s41467-025-67582-y)
Supplement: Supplementary file 1 — Supplementary Information [file 41467_2025_67582_MOESM1_ESM.pdf]

Dual phase high temperature Si_3N_4/Al(Ti)N films with tunable thermal conductivity

Zhaohe Gao[a,b,c*], Han Liu[a], Jinchi Sun[d], Justyna Kulczyk-Malecka[e], Xiaodong Liu[a], Etienne Bousser[f], Peter Kelly[e], Yu-Lung Chiu[b], Philip J. Withers[a], Ping Xiao[a]

[a]Henry Royce Institute, Department of Materials, University of Manchester, Manchester, M13 9PL, UK
[b]School of Metallurgy and Materials, University of Birmingham, Birmingham, B15 2TT, UK
[c]Centre for Manufacturing and Materials, Coventry University, Coventry, CV1 5FB, UK
[d]Department of Materials Science and Engineering and Materials Research Laboratory, University of Illinois at Urbana-Champaign, Urbana, IL 61801, USA.
[e] Surface Engineering Group, Manchester Fuel Cell Innovation Centre, Manchester Metropolitan University, Manchester M1 5GD, UK
[f]Department of Engineering Physics, Polytechnique Montréal, Montreal, H3T 1J4, Canada

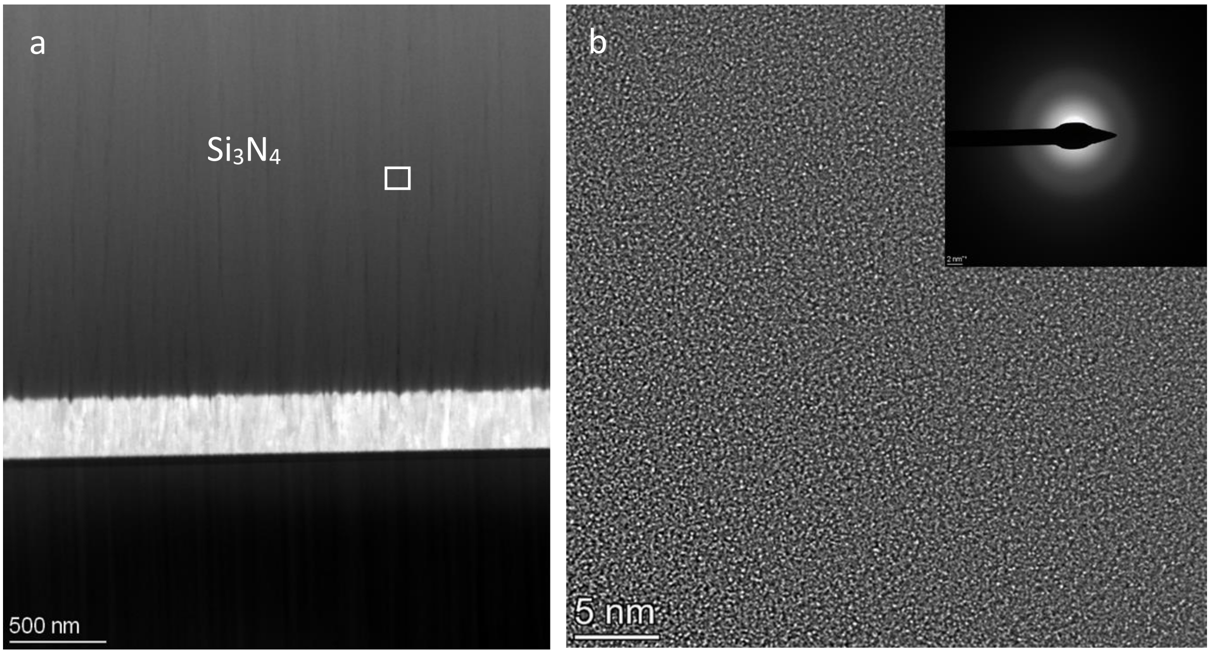

Fig.s1 Microstructure of as-deposited Si_3N_4 coating revealed by TEM. (a) Cross-sectional HAADF image of as-deposited Si_3N_4 coating with an Mo interlayer on Si wafer; (b) HRTEM image of as-deposited Si_3N_4 coating with diffraction pattern of the Si_3N_4 inset, acquiring from the rectangle box in (a).

Corresponding authors: Z.Gao (Current address: Materials Genome Institute & State Key Laboratory of Advanced Refractories, Shanghai University, Shanghai, 200444, China. gaozhaohe2013@gmail.com or z_gao@shu.edu.cn), Y.C. (y.chiu@bham.ac.uk), P.X. (p.xiao@manchester.ac.uk)

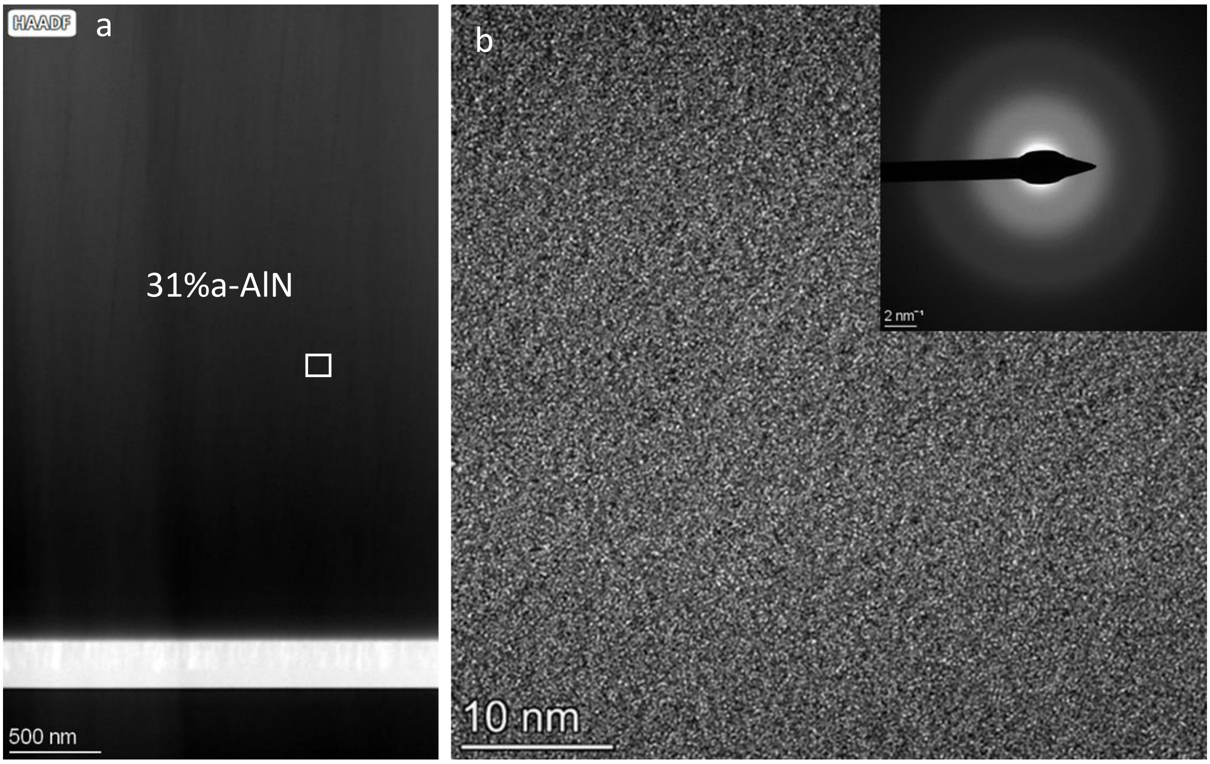

Fig.s2 Microstructure of as-deposited 31%a-AlN coating revealed by TEM. (a) Cross-sectional HAADF image of as-deposited 31%a-AlN with Mo interlayer on Si wafer; (b) HRTEM image of as-deposited 31%a-AlN coating with diffraction pattern of the 31%a-AlN inset, acquired from the rectangular box in (a).

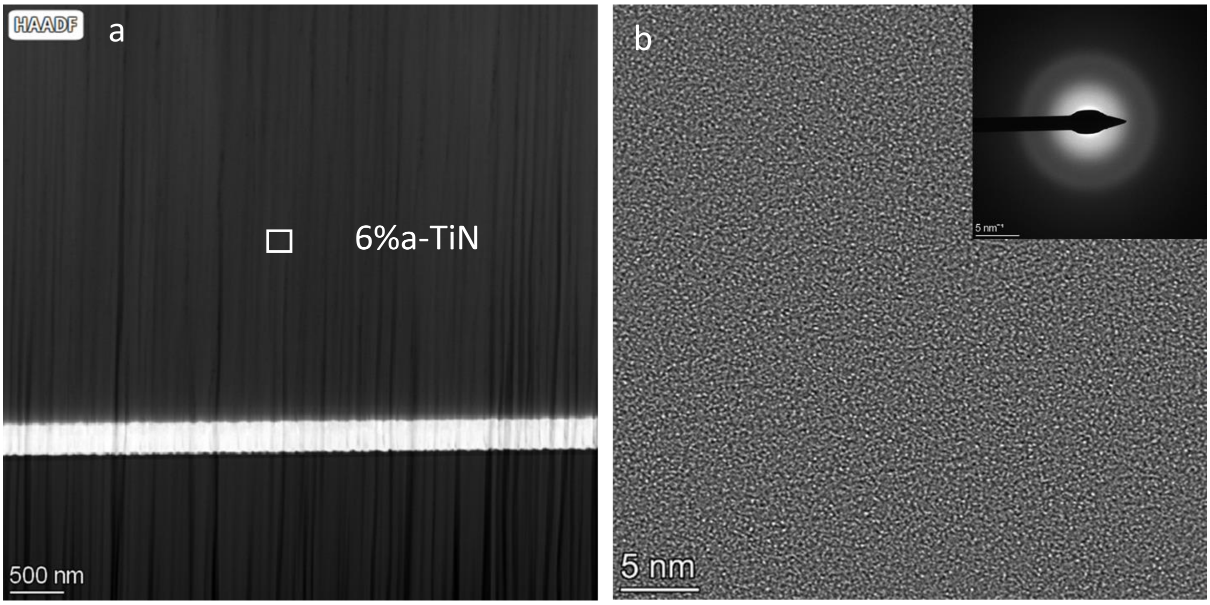

Fig.s3 Microstructure of as-deposited 6%a-TiN coating revealed by TEM. (a) Cross-sectional HAADF image of as-deposited 6%a-TiN coating with Mo interlayer on Si wafer; (b) HRTEM image of as-deposited 6%a-TiN coating with diffraction pattern of the 6%a-TiN inset, acquired from the rectangular box in (a).

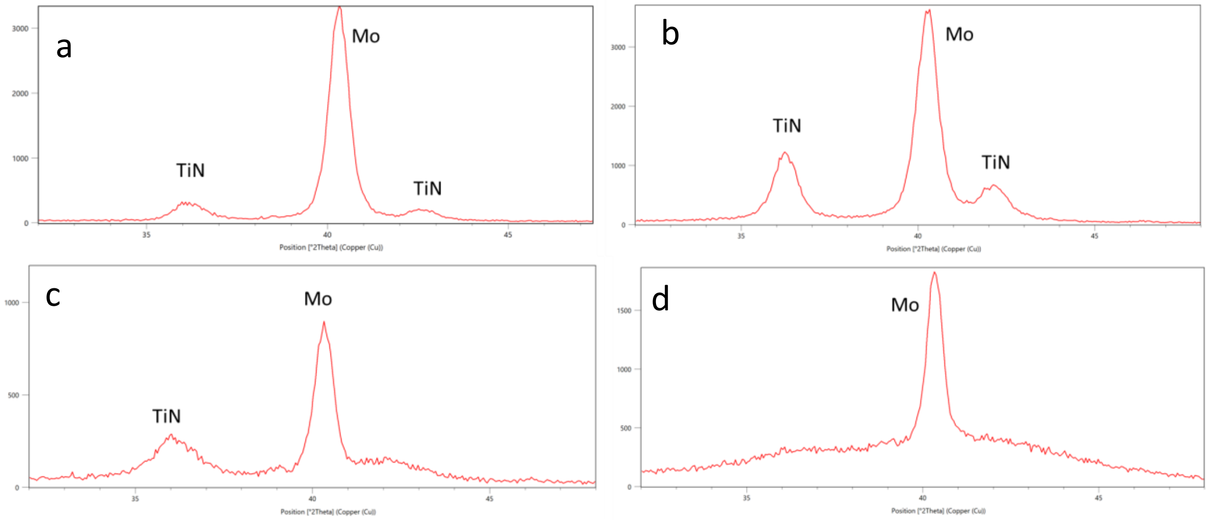

Fig.s4 GIXRD pattern of as-deposited (a)TiN, (b) 88%c-TiN, (c) 81%c-TiN, (d) 70%a-TiN coatings with Mo interlayers on Si wafer.

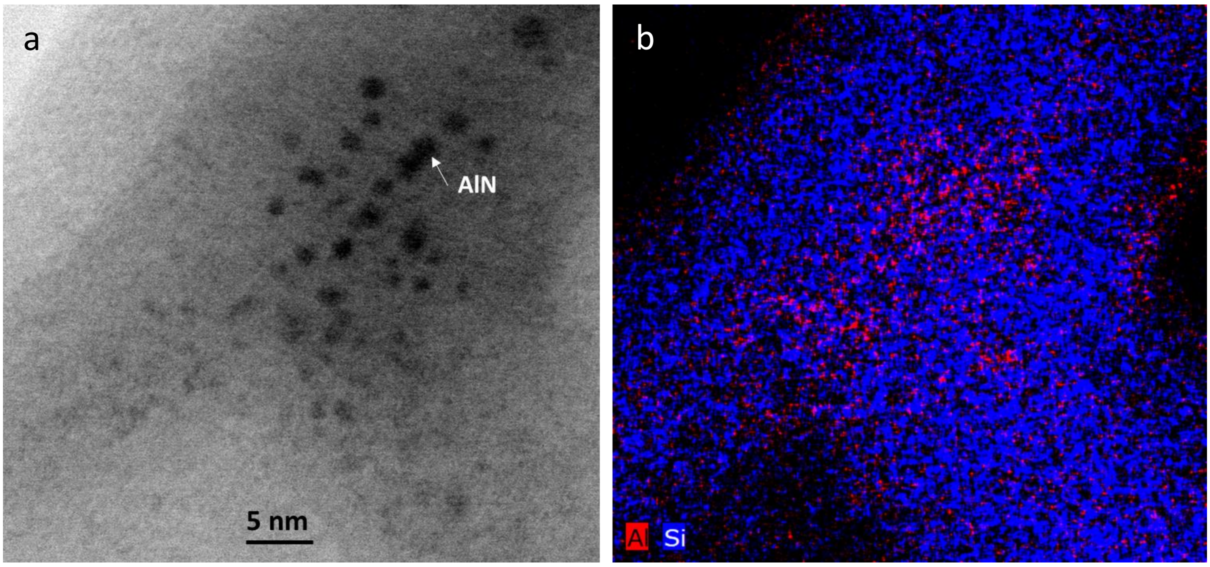

Fig.s5 Microstructure and element distribution of 15%a-AlN coating. (a) Cross-sectional STEM BF image of as-deposited 15%a-AlN coating by an aberration-corrected TEM and (b) corresponding EDS Si and Al elemental maps.

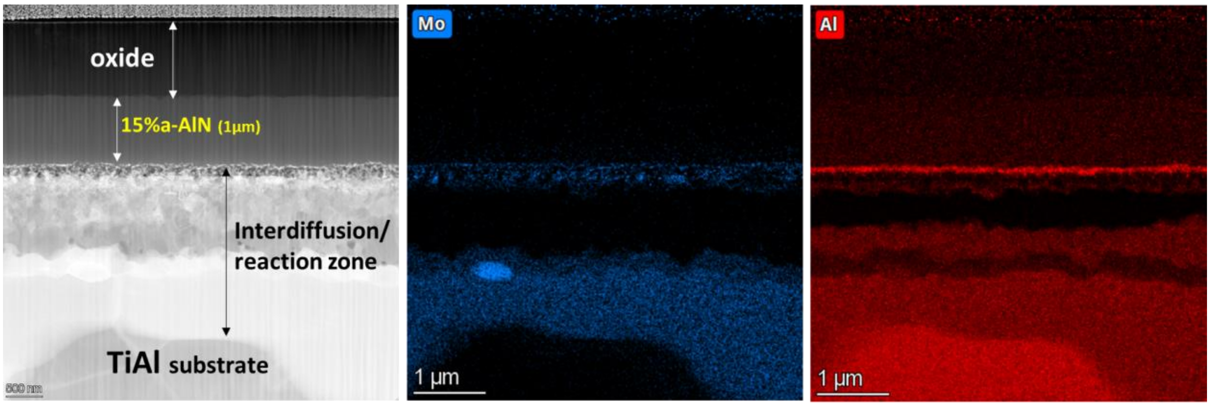

Fig.s6 Microstructure and elemental distribution of 15%a-AlN (a-Si_3N_4/a-AlN) coating after thermal exposure at 900°C for 100 h.

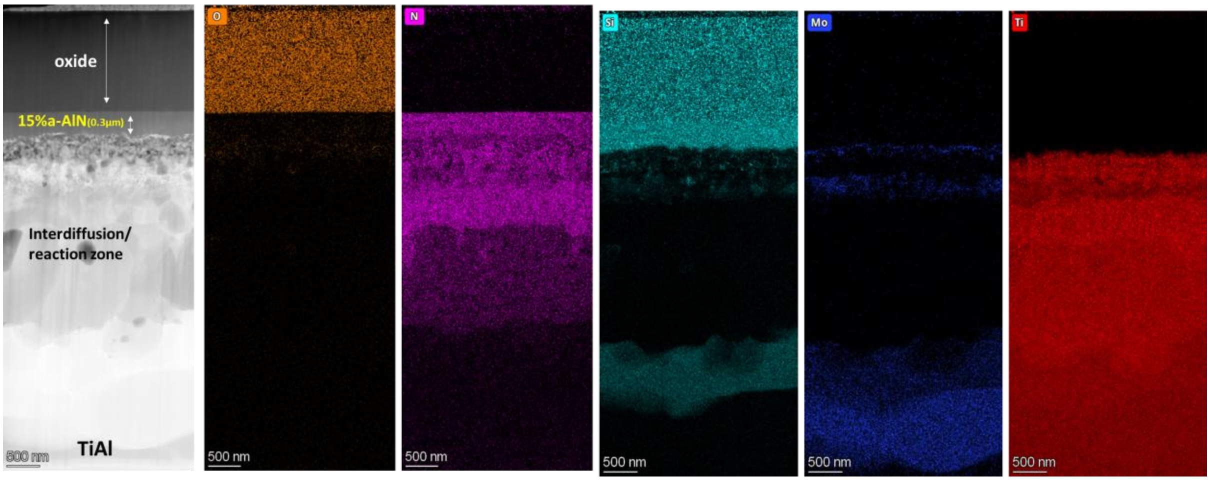

Fig.s7 Microstructure and elemental distribution of 15%a-AlN (a-Si_3N_4/a-AlN) coating after thermal exposure at 1000°C for 50 h.

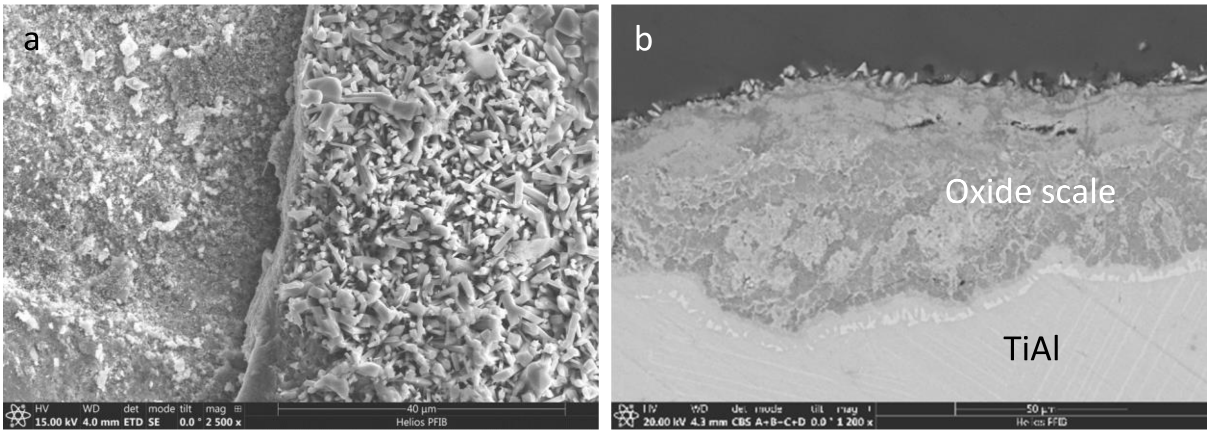

Fig.s8 SEM microstructure of bare TiAl after thermal exposure to 900°C for 100 h. (a) Top surface SEM observation; (b) Cross-sectional SEM observation, indicating a thick layer of oxide scale.

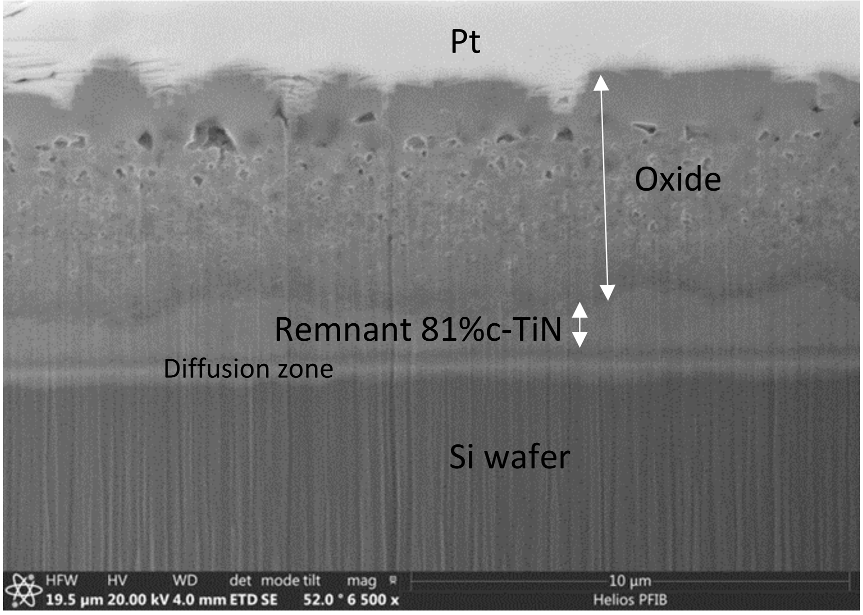

Fig.s9 Cross-sectional SEM microstructure of 81%c-TiN (a-Si_3N_4/c-TiN) after thermal exposure to 900°C for 50 h, indicating a 6-7 µm thick layer of oxide scale. The thickness of as-deposited 81%c-TiN is 7.7 µm.
